# Influence of Coil Power Ranges on the E-Liquid Consumption in Vaping Devices

**DOI:** 10.3390/ijerph15091853

**Published:** 2018-08-28

**Authors:** Sébastien Soulet, Marie Duquesne, Jean Toutain, Charly Pairaud, Hélène Lalo

**Affiliations:** 1 Laboratoire Français du E-Liquide (LFEL), 218 Avenue du Haut Lévêque, 33600 Pessac, France; charly.pairaud@lfel.fr (C.P.); helene.lalo@lfel.fr (H.L.); 2 Université de Bordeaux, CNRS, I2M Bordeaux, Site ENSAM, Esplanade des Arts et Métiers, 33400 Talence, France; 3 Bordeaux INP, CNRS, I2M Bordeaux, Site ENSAM, 16 Avenue Pey Berland, 33600 Pessac, France; marie.duquesne@enscbp.fr (M.D.); jean.toutain@enscbp.fr (J.T.)

**Keywords:** electronic cigarette, vaping devices, emission generation, standardization, atomizer, supplied power

## Abstract

As electronic cigarettes (e-cigarettes) represent a new constantly evolving product category, the systematic analysis of the developed devices and the e-liquid vaporization is challenging. Indeed, understanding how e-cigarettes work and the role of key parameters in the process are major issues. This work focuses on an experimental study of how the power supplied by the battery to the atomizer coil influences e-liquid consumption. The reproducibility and the repeatability of e-liquid consumption were investigated over 20 series of 20 puffs for one of the tested atomizers. Then, the reproducibility and the repeatability of the e-liquid consumption was investigated over five series of 20 puffs for each tested atomizer. The wire behavior according to the supplied power could be separated into three regimes: under-heating (insufficient power to generate an aerosol), optimal vaporization characterized by a linear trend (vaporization of the e-liquid proportional to the supplied energy) and over-heating (dry-burn occurs). Using a controllable and repeatable energy supply, the reproducibility of the quantity of vaporized e-liquid was verified for each of the five series of 20 puffs programed for all the atomizers except one. Finally, the influence of the supplied power on the vaporization and the consumption of the e-liquid as well as the optimal power ranges were investigated and discussed. The results showed that atomizers with resistance ranging from 1 Ω to 1.8 Ω are efficient using all the energy supplied by the battery to vaporize the e-liquid and reducing the energy lost in the cotton or in the metal part of atomizer coil.

## 1. Introduction

The electronic cigarette (e-cigarette or e-cig) concept was developed in the early 1960s by Gilbert and patented in 1965. His invention produced a nicotine-free aerosol containing flavor [1] in order to replace tobacco smoke. This device was then forgotten until 2001. Hon Lik, a Chinese pharmacist and traditional medicine expert, worked on this concept and developed his first prototype based on an ultrasonic emitter and a pressurized liquid. At its activation, the emitter generates high frequency waves and the resulting vibration allows separating the e-liquid into thin particles. This prototype was patented in 2003 [2,3] and was given up in favor of a device composed of a heating element generating aerosol. Since, this kind of atomizer design—mainly composed of a battery, a resistive wire, a fibrous medium (wick), and a multi-component liquid mixture (e-liquid)—has spread widely in the market. Currently, more than 500 e-cigarette manufacturers are present in the market creating an important device variety [4]. They provide users with a wide range of products and ability to adjust the equipment as they desire.

However, this fast-emerging market faces many public health questions. The major one is linked to the safety of the products compared to tobacco cigarettes. Current evidence attests to risk reduction for smokers who switch to vaping [5,6,7,8]. Indeed, researchers have compared the influence of e-cigarettes and supplied power [9,10,11,12,13], e-liquid composition [14], and user topography [15] on nicotine delivery and generated by-products. The quantity of toxic aldehydes generated in the aerosol while lower than in cigarette smoke, could be reduced by adjusting the type of device and the vaporization conditions. For example, in dry hit—a term used to describe when an atomizer coil receives a current with a wick which is not completely wet by the e-liquid; in which case a burning sensation could be experienced by the user due to a fast and important increase in the wire temperature leading to a degradation of the wick—conditions, when the wick is dried and the heating coil continues to heat up, the generated by-products are significantly increased to be closer to, although still less than, levels in tobacco smoke [16,17]. However, under realistic conditions, users are not chronically exposed to these high levels because the user feels a strong taste of burning and stops vaping.

In addition to public health questions, the factors influencing e-cigarette performance must also be investigated. They are complex and include, but are not limited to: heat and mass transfers in a cylindrical fibrous medium impregnated with a multi-component e-liquid, vaporization of multi-component systems. Therefore, the systematic analysis of the devices and the e-liquid vaporization is challenging. Thus, understanding how the e-cigarettes work and the influence of the key parameters influencing their performance have become major issues in this sector. Indeed, the e-liquid consumption informs e-cigarette performance and the optimal use conditions. For now, we identified three categories of parameters influencing e-liquid consumption: parameters related to the design of the atomizer (coil design, supplied power), parameters related to e-liquids (composition), and parameters related to the user (inhalation profile).

This paper only focuses on the influence of the atomizer design and its power range on e-liquid consumption. The two other categories of influence will be addressed in two further publications. Therefore, for this study, only one e-liquid and only one inhalation profile were used as references and tested with 13 commercial atomizers. We first studied the reproducibility of e-liquid consumption over a series of tests for each tested device. Once this reproducibility was determined, we investigated the influence of power and the design of the atomizer on the e-liquid consumption.

## 2. Materials and Methods

**Figure 1:**
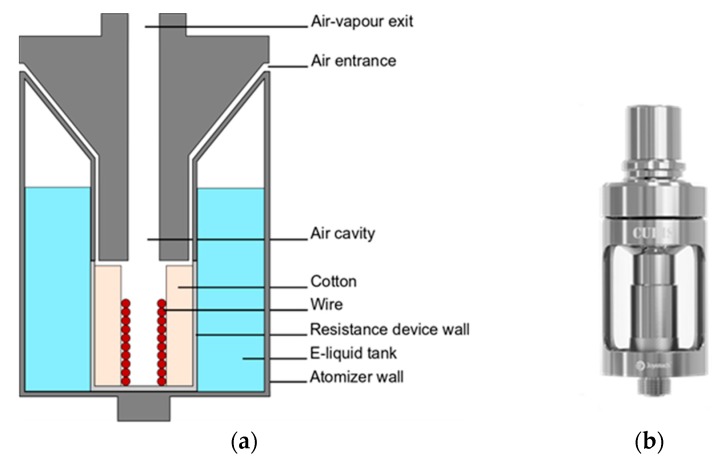
(**a**) Sectional scheme and (**b**) picture of the Cubis atomizer.

The principle of an e-cigarette is simple and common to all designs: a battery supplies a current to the coil wire that heats a wick soaked with a liquid, called e-liquid, allowing its vaporization and the formation of an aerosol (see Figure 1).

These components are described in the following subsections.

### 2.1. E-Liquid

Usually, the vaporized e-liquid mainly contains propylene glycol (PG) and vegetable glycerin (VG). It can also contain, according to the consumer’s desires: nicotine, water, alcohol, and flavor compounds. The liquids tested in this study are listed in Table 1.

The e-liquid used in this study was a quaternary mixture of the liquids in Table 2. This e-liquid was the only mixture tested in this study and will be used as a reference to evaluate the influence of vaporizing parameters on vaporization and therefore, on its consumption.

### 2.2. Atomizers

Each tested atomizer is made of a coil and wick. The coil is composed of a rolled wire. This wire is composed of three parts: both extremities are non-resistive (to avoid the burning of the closest elements they are in contact with) and the central part, connected to both non-resistive extremities, is resistive (to insure an optimal heating of the soaked wick). The wick is rolled around this coil.

We tested 13 commercial coils: Cubis (0.5, 1, and 1.5 Ω) and Unimax (0.4 Ω) from Joyetech^TM^, CL Tank (0.5 Ω) and Mini-C (0.5 Ω, 1.5 Ω) from Kangertech^TM^, T18 (1.5 Ω) and I-Sub (0.5 Ω) from Innokin^TM^, Nautilus (1.8 Ω) and K3 (1.8 Ω) from Aspire^TM^, GS Air (1.5 Ω) and Melo III (0.4 Ω) from Eleaf^TM^. Each commercial atomizer was tested with three exemplars in order to measure variability over the devices. The atomizers are listed and detailed in Table 3.

In this work, we will experimentally study the influence of the atomizer design and of the supplied power on e-liquid consumption. As we are studying commercial atomizers, the coil devices are closed (one piece containing an unreachable wire and wick). Therefore, we could not control the geometrical dimensions of the wire or of the wick. We can only get a rough idea of them by opening (i.e., destroying) the closed coil, removing and unrolling the wire and the wick after our experiments were completed. For these reasons, we considered not only the wire but rather the assembly (wire + wick) in this study.

### 2.3. Vaping Machine

So far, e-cigarettes have always been tested on smoking machines, adapted for the testing of vaping products. The vaping machine Universal System for Analysis of Vaping (U-SAV), the first vaping machine especially designed to test e-cigarettes was designed and developed by LFEL (French Laboratory of E-liquid). Indeed, LFEL designed its own vaping machine to control all the physical parameters influencing the e-liquid vaporization: power, resistance, flow rate, inhalation time, atomizer, etc. In a previous publication [18], the performed experiments proved the reproducibility and repeatability of U-SAV in air-flow profile and supplied power generation. The e-liquid consumption reproducibility and repeatability were also checked over experiments using a standardized e-liquid and protocol. Then, it was illustrated how the supplied power has a significant impact on the e-liquid consumption and due to their discharge issues and inaccurate power regulation batteries should be avoided in emissions generation for their analysis. U-SAV protocol is based on the AFNOR (French Association for Standardization) protocol [19]. The manipulation is composed of 100 puffs divided in five series of 20 puffs [19]. Each series was separated by a 5-min-break referred to inter-series. In agreement with the ISO (International Organization for Standardization) standard [20], each puff has a period of 30 s including 3 s of vaporization and 27 s of rest. The flow rate is programmed at 18.3 mL·s^−1^. The atomizer inclination was set at 45° during the vaporization process and back to 0° with respect to vertical position, during 10 s, between two series in order to simulate the user’s behavior as defined in the (AFNOR) standard.

The built-in energy supply of U-SAV is used in order to avoid problems from battery discharges. The programed power regulation takes into account the wire resistance rise with its increase in temperature. Measuring the real-time electric current and voltage (i.e., the supplied power real-time measurement) and considering the increase in resistance, the machine regulates the voltage instruction to stick to the selected power. Therefore, U-SAV is a practical tool ensuring the stability of the required power and avoiding the power fluctuations due to battery discharges (which could impact on the quality of the regulation). The tested power ranges are those given by the manufacturers (Table 3).

### 2.4. Experiments

The influence of each parameter was characterized by weighting the tested atomizer before and after each experiment. Then, we calculated the difference in the two masses to obtain the mass of vaporized e-liquid. This value is finally divided by the number of puffs during the experiment. The Mettler AT261 Delta Range laboratory scale (Mettler-Toledo GmbH, Greifensee, Switzerland) range is 1 mg to 205 g with a precision of 0.1 mg.

A first series of experiments aimed to test the reproducibility and the repeatability of the e-liquid consumptions over identical and successive series of tests for only one atomizer. The repeated experiment was conducted with the Cubis 1 Ω atomizer. This device was defined as the reference for emission generation in LFEL laboratory and consequently for the further studies mentioned in the introduction (influence of e-liquid composition and inhalation profile on the e-liquid consumption). For these reasons, we characterized this atomizer performance over a larger protocol than the one defined in the AFNOR standard. It was filled with the reference e-liquid up to the maximum line required by the manufacturer. Five series of 20 puffs have been programed on U-SAV vaping machine (see previous subsection). The power setting was 15 W. This reproducibility test was stopped when a burnt smell was perceived and a visual aerosol change occurs from a white dense to a transparent bluish aerosol. Besides, we opened the closed coil and observed the partially burnt wick (black streaks characterizing the beginning of combustion) after our experiments.

A second series of experiments was performed to observe the e-liquid consumption reproducibility over the five series of 20 puffs required by the AFNOR standard at a reference power for each atomizer. As the user’s behavior is unpredictable in terms of the power applied, we selected an arbitrary power value for each atomizer and kept it as a reference for all the experiments. This ‘arbitrary’ power was selected by asking a small sample of e-cigarette users to test out the device and tell us the power setting according to their preference. The atomizers were weighted after the end of each series (during inter-series).

A final series of experiments was performed to evaluate the influence of the supplied powers and the power ranges on the e-liquid consumption. The first step of this series consists of determining the power range of optimal vaporization. Once this optimal power range is identified for each of the tested atomizers, each range was divided to obtain five measurements, with uniform distribution, by the atomizer (or four when the last tested power gave burning results).

## 3. Results

This section aims to present the influence of the devices’ design and vaporizing parameters (resistance, power etc.) on the e-liquid consumption. First, the reproducibility and the repeatability of the obtained mass of vaporized e-liquid at a constant power is investigated and discussed. Then, the optimal power range is identified and the corresponding e-liquid consumption is measured for each of the 13 tested vaping devices.

### 3.1. Reproducibility over Series

**Figure 2:**
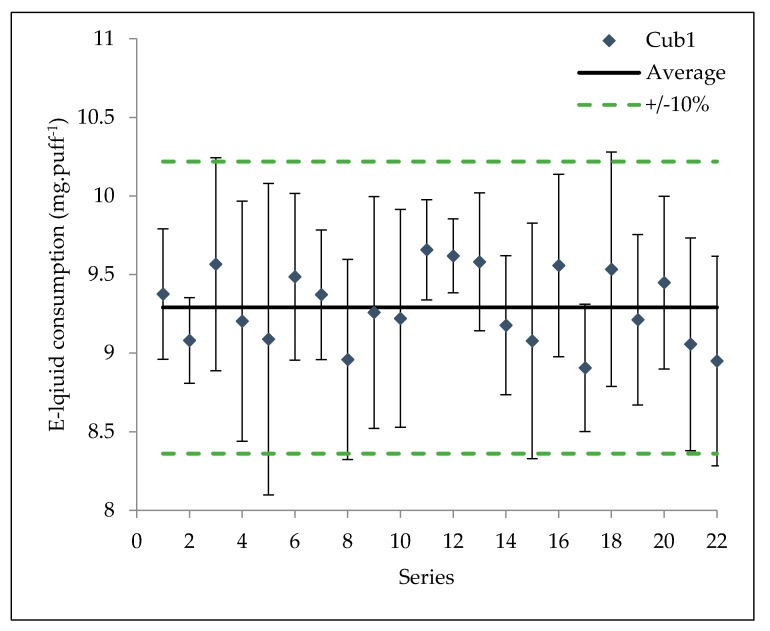
E-liquid consumptions—repeatability of the average e-liquid consumption over 22 series for the Cub1 atomizer.

The influence of the number of performed series on e-liquid consumption is illustrated in Figure 2. The average e-liquid consumption by performed series is quite constant over each series. The mass of vaporized e-liquid, i.e., the lost mass of e-liquid within the atomizer, by series presents an average value of 9.29 mg·puff^−1^ with a standard deviation of ±0.24 mg·puff^−1^ over 22 series. After the last of 22 series, during the weighing of the atomizer, a burnt smell was noticed. This smell became strong at the 23rd series during which no aerosol left the drip-tip. This means that, under the same operating conditions, the measured mass of vaporized e-liquid is stable and repeatable over 22 series. This observation let us assume that the wick is automatically wet between two puffs and no burning feeling might be measured during the experiment.

The reproducibility and repeatability are validated over 20 series for one of the tested vaping devices (Cub1). The reproducibility of the 13 different commercial atomizers over five series is tested at a selected reference power (Table 4).

**Figure 3:**
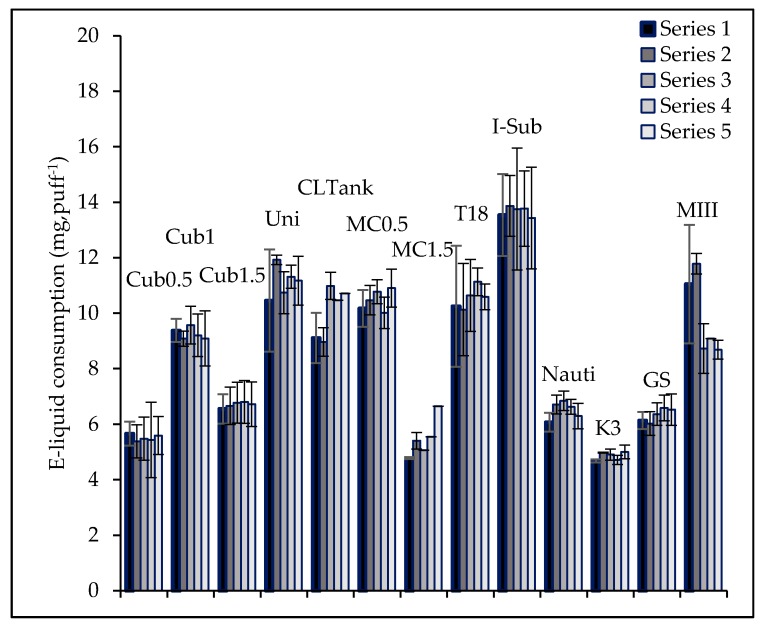
E-liquid consumption—repeatability of the average e-liquid consumption over five series for the 13 atomizers.

Each atomizer has its own average mass of vaporized e-liquid per series (Figure 3) ranging from 4.86 mg·puff^−1^ for K3 to 13.67 mg·puff^−1^ for I-Sub. 12 over the 13 presented atomizers have a low deviation over the five series (Table 4). MIII atomizer has a high deviation between series. E-liquid is more vaporized at the beginning of the series than at the end.

### 3.2. Power Ranges

**Figure 4:**
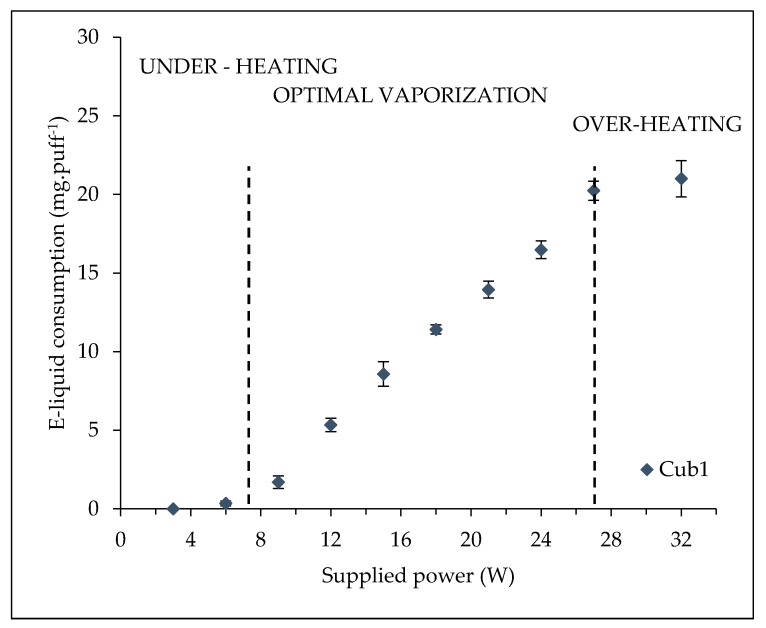
Influence of the supplied power on the mass of e-liquid vaporized for the Cubis atomizer 1 Ω from 3 to 32 W.

The power influence on e-liquid consumption could be divided to three regimes (see an example with the Cub1 atomizer in Figure 4). From 0 to 7 W, the supplied power is not sufficient to vaporize all the e-liquid; therefore just a slight amount of e-liquid is vaporized. We call this regime ‘under-heating’, and during it, the power was not a significant influence on the amount of vaporized e-liquid. The associated curve is almost horizontal and the corresponding slope is close to zero. From 8 to 27 W, e-liquid consumption is quite linearly linked to the supplied power with a significant slope. We call this second regime optimal vaporization. Regarding powers above 27 W, e-liquid consumption is approximately equal to the one at 27 W (trend almost horizontal). Indeed, the power is exceeding the power required to vaporize the whole e-liquid contained in the wick. Therefore, above 27 W, there is no additional influence of an increase in power on the e-liquid consumption. We call this regime over-heating.

Thus, we will focus on the optimal vaporization regime, because it appears to be the only one having a strong influence on e-liquid consumption. Talih et al. [21] developed a simplified steady state model of nicotine flux and showed that under certain conditions, the vaporization rate during a puff may be expressed as a linear function of the supplied power with a slope proportional to the inverse of the mass enthalpy of vaporization. Further work will consist in developing a more precise model considering the compositions, the temperature and the pressure dependency on all the phases properties that impact on the energy required to vaporize the e-liquid.

**Figure 5:**
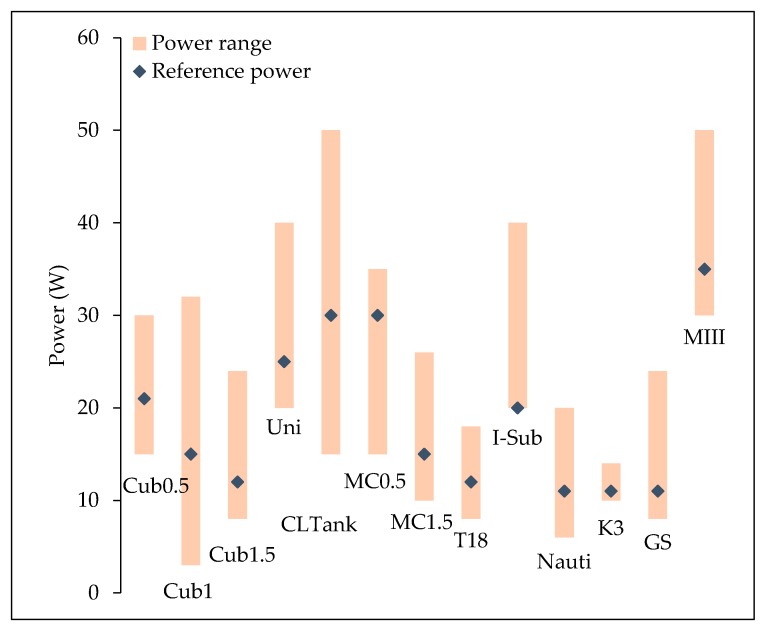
Some power ranges and the arbitrary reference power.

In the meanwhile, based on [21] and as it is coherent with the standard deviations of the measurements (see Figure 4), we assume that this optimal vaporization regime presents a quite linear trend with a slope depending on the vaporization enthalpy (*H^v^*) of e-liquid.

(1) $$\;MEV=bP+c\;with\;b\;\alpha \frac{1}{H^{v}\;}$$

where *MEV* is the mass of e-liquid vaporized in mg and b is express in mg·puff^−1^·W^−1^ (same dimension with g·J^−1^). This regime and the associated power ranges are determined for each tested device (Table 5). The localization of their arbitrary reference power in this range is represented in Figure 5 for each studied device.

Each device had an optimal regime which was usually close to the power range of use requirements given by the manufacturer. In this regime, e-liquid consumption is expressed as a linear function of the supplied power. It means that to be able to determine e-liquid consumption for a given power and a precise vaping device, we calculated the b and c coefficients mentioned in Equation (1), their respective standard deviations, Δb and Δc, as well as the corresponding coefficient of determination, *R*². The latter was computed using Equation (2)

(2) $$\;R^{2}={\left[\frac{\sum_{n}\left(P-\bar{P}\;\right)(MEV-\bar{MEV)}}{\sqrt{\sum_{n}{\left(P-\bar{P}\right)}^{2}\sum_{n}(MEV-{\bar{MEV)}}^{2}}}\right]}^{2}$$

with $\bar{P}$ being the average value of *P* and $\bar{MEV}$ the average value of the mass of e-liquid vaporized (MEV) over the *n* measurements.

The obtained results and the power ranges are given for each studied device in Table 6.

Ten atomizers have *R*² superior to 0.99, 2 between 0.97 and 0.99. T18 is the only device that had a low *R*² (0.9277).

The 13 commercial atomizers were tested characterized by different resistance values and could be separated in two main categories, also representative of two categories of e-cigarette users.

**Figure 6:**
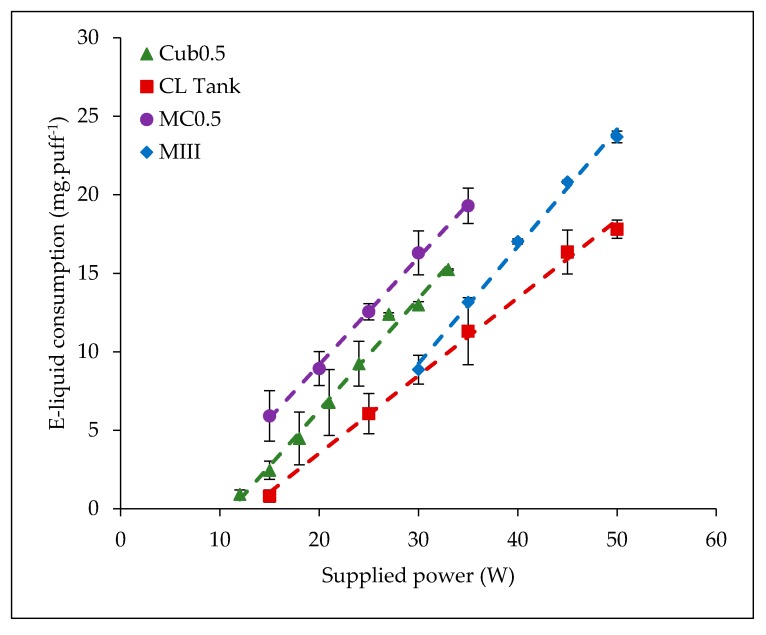
E-liquid consumptions versus supplied power—examples of the optimal vaporization regime of atomizers with 0.5 Ω coils for users with a direct inhalation profile.

**Figure 7:**
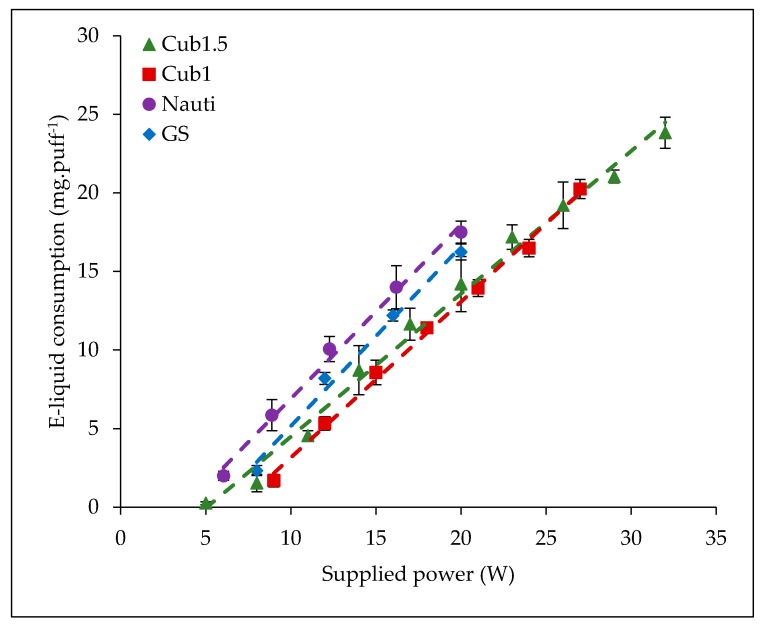
E-liquid consumptions versus supplied power—examples of the optimal vaporization regime of atomizers with coils from 1 Ω to 1.8 Ω for mouth or mouth to lungs inhalation profiles close to the ISO 20768 standard one.

- Six atomizers with a resistance of 0.5 Ω allow supplying power above 25 W (MIII, I-Sub, MCO.5 Ω, Uni, CLTank and Cub0.5). Their b-coefficient presents an average value of 0.73 mg·puff^−1^·W^−1^ ± 0.12 mg·puff^−1^·W^−1^. Some examples of the obtained trends for this group are presented in Figure 6. This first category of atomizers allows high supplied powers (e.g., the maximum power delivered by CLTank is 60 W and 100 W by MIII). These atomizers are designed to generate a high quantity of aerosol for users with a direct lung inhalation profile. This profile is characterized by high inhaled volumes (closer to the current pulmonary volume), longer puff durations (greater than 4–5 s [22]), and high flow-rates (greater than 50–66 mL·s^−1^). This means that these atomizers are designed with low resistance air-flows.
- Seven atomizers with a resistance ranging from 1 Ω to 1.8 Ω allowed power superior to 5 W but which do not exceed 25 W (Cub1.5, T18, Nauti, K3, GS, Cub1, and MC1.5 Ω). Their b-coefficient presents an average value of 1.09 mg·puff^−1^·W^−1^ ± 0.07 mg·puff^−1^·W^−1^. Some examples of the obtained trends for this group are presented in Figure 7. This second category of atomizers is used at low supplied powers and was initially designed for lower aerosol generation. Indeed, users of these atomizers present a mouth or mouth-to-lung inhalation profile. This term is used to describe people who fill their mouth of the aerosol and inhale it with or without an air dilution before. This is characterized by a low volume of aerosol inhaled (more or less the mouth volume), small puff duration and low flow rate value. This type of profile is close to the one defined as a reference in the ISO 20768 Standard. This kind of atomizer has highest air-flow resistance in order to limit intense inhalation by the user.

B-coefficient average values for these two groups are different: only 0.93 W are used for the second group versus 1.37 W (32% more) to vaporize the same mass of e-liquid (1 mg·puff^−1^). The second group of atomizers seems then to be more efficient under the studied operating conditions because it allows only using the power required to vaporize e-liquid and consequently allows reducing thermal losses in the atomizer. This difference between the two groups of atomizers will be discussed in next section.

## 4. Discussion

First, the T18 atomizer presents a low coefficient of determination (*R*² = 09277). Additional experiments must be performed to refine the corresponding results.

In a thermal point of view, the second group of atomizers, tested with an appropriate inhalation profile, have close b-coefficients which is consistent with Talih’s work [21]. They propose a relation linking the mass flow of e-liquid vaporized and the supplied power by the e-liquid mass enthalpy of vaporization (*H^v^* in J·mg^−1^). An estimation of the mass enthalpy of vaporization for the tested e-liquid (composed of n pure components) could be calculated using Equation (3)

(3) $$\;H^{v}=\frac{\sum_{i}^{n}x_{i}h_{i}^{v}\;}{\sum_{i}^{n}x_{i}M_{i}}*10^{-3}$$

with $h_{i}^{v}$ being mass enthalpy of vaporization, *M_i_* being the molar mass of component *i*, and *x_i_* being the mole percent of *i* pure component. Using data from Table 2, we obtained an enthalpy of vaporization of 0.933 J·mg^−1^ which is very close to the inverse of the average b-coefficient (1/b = 0.920 J·mg^−1^). It means that almost all the supplied energy by the battery is used to vaporize the e-liquid and that these atomizers are very efficient with reduced thermal losses which is not the case for the first group. Although it is the same tested e-liquid and should have the same enthalpy of vaporization, the inverse of the average b-coefficient is far from the one expected (1/b = 1.408 J·mg^−1^).

The second group of atomizers being more efficient than the first one is in contradiction with what was expected. This observation might be due to the inhalation profile used during the experiments. Indeed, this low profile of inhalation (3-s puff duration, 18.3 mL·s^−1^ and 55 mL) is not intended for atomizers generating high quantity of aerosol. Results presented in this paper might be completely different with the use of an intense profile. This latter profile should be more realistic for direct-inhalation users. However, as there is currently only one standardized profile, we characterize the device using this maladjusted profile.

Talih et al. also illustrated that the quantity of nicotine in the emission could reliably be predicted from measurement of the mass of e-liquid vaporized and knowledge of the nicotine concentration of the liquid used. Based on their observation, the repeatability and reproducibility of e-liquid consumption over series, shown in Figure 3, let us suppose that the nicotine consistency of the tested device is checked. Consequently, nicotine consistency required by the Tobacco Product Directive could be simply verified with a mass of e-liquid vaporized analysis over series.

## 5. Conclusions

This paper focuses on the study of the influence of supplied powers and atomizer designs on e-liquid consumption. The studied e-liquid was a quaternary mixture made of 0.2% of nicotine, 6.4% of ethanol, 42% of propylene-glycol, and 51.4% of vegetable glycerin (% in mass). Thirteen commercial coils were tested and user behavior was simulated using U-SAV, a vaping machine.

The reproducibility and the repeatability of e-liquid consumption were verified over 22 series of 20 puffs for one of the tested atomizers (Cub1). The mass of vaporized e-liquid per series presents an average value of 9.29 mg·puff^−1^ with a standard deviation of ± 0.24 mg·puff^−1^ over 22 series. Then, the stabilities of the 13 different commercial atomizers were tested over five series. Each atomizer has its own average mass of vaporized e-liquid per series ranging from approximatively 5 mg·puff^−1^ for K3 to 14 mg·puff^−1^ for I-Sub. All the atomizers, except one, have a low deviation over the five series and MIII atomizer has a high average e-liquid consumption variation between the first and last series.

Seven of the 13 atomizers present close slope coefficients 1.09 mg·puff^−1^·W^−1^ ± 0.07 mg·puff^−1^·W^−1^ (i.e., 1/b= 0.920 J·mg^−1^) corresponding to the mass enthalpy of vaporization of the tested liquid of 0.933 J·mg^−1^. This observation is consistent with the linear relation between e-liquid consumption and the supplied power proposed by Talih et al. [21]. The six other devices produced lower slope-coefficients and a different behavior. Although the enthalpy of vaporization is an intrinsic property of the studied liquid and the e-liquid being the same for all experiments, the slope is not the same for all the atomizers. This means that, for some atomizers, the assumptions made about them might be revised and the simplified linear model is not sufficient to represent the real behavior of the atomizer and the liquid inside it.

For this study, a single e-liquid and only one inhalation profile were used as references and tested with 13 commercial atomizers. Further work will report on the influence of e-liquid composition and inhalation profile on e-liquid consumption, this time using Cub1 coil as atomizer reference.

A more intense profile of inhalation shall be standardized and defined in accordance with the typical user’s profile for low resistance atomizers (high quantity of generated vapor) as the ones used in this publication.

## Figures and Tables

**Table 1:** List of the studied pure liquids

	Acronym	CAS Number	Formula	Provider	Purity (%)
Nicotine	Nico	54-11-5	C_10_H_14_N_2_	ALCHEM	≥99.2%
Ethanol	EtOH	64-17-5	C_2_H_6_O	GROSSERON	96%
1,2-propanediol	PG	57-55-6	C_3_H_8_O_2_	BRENNTAG	≥99.8%
1,2,3-propanetriol	VG	56-81-5	C_3_H_8_O_3_	AMI CHIMIE	99.5%

**Table 2:** Composition and properties of the studied liquid given at ambient temperature (Design Institute for Physical Properties (DIPPR) database: https://www.aiche.org/dippr).

Quaternary Mixtures	Volume Percent (%)	Density (g·cm^−3^)	Mass Percent (%)	Molar Mass (*M_i_* in g·mol^−1^)	Mole Percent (*x_i_* in %)	Molar Enthalpy of Vaporization ($h_{i}^{v}\;\mathrm{in}\;\mathrm{kJ}\text{·}\mathrm{mol}$^−1^)
Nicotine (Nico)	0.20	1.01	0.18	162.24	0.09	56.6
Ethanol (EtOH)	10.0	0.79	7.09	46.07	12.24	42.847
1,2-propanediol (PG)	44.8	1.04	41.83	76.10	43.71	66.980
1,2,3-propanetriol (VG)	45.0	1.26	50.90	92.09	43.96	90.214

**Table 3:** Manufacturer general information about the studied devices

Manufacturer	Reference	Resistance	Metal	Wick	Notation	Min	Max
Joyetech	Cubis	0.5 Ω	SS316L	Organic cotton	Cub0.5	15 W	30 W
	Cubis	1 Ω	SS316L	Organic cotton	Cub1	10 W	25 W
	Cubis	1.5 Ω	Kanthal ^1^	Organic cotton	Cub1.5	8 W	20 W
	Unimax	0.5 Ω	Kanthal	Organic cotton	Uni	20 W	40 W
Kangertech	CL Tank	0.5 Ω	SS316L	Organic cotton	CLTank	15 W	60 W
	Mini C	0.5 Ω	Kanthal	Ceramic	MC0.5	15 W	30 W
	Mini C	1.5 Ω	Nichrome	Organic cotton	MC1.5	10 W	26 W
Innokin	T18	1.5 Ω	Kanthal	Organic cotton	T18	8 W	14 W
	I-Sub	0.5 Ω	Kanthal	Organic cotton	I-Sub	20 W	35 W
Aspire	Nautilus	1.8 Ω	Kanthal ^1^	Cotton	Nauti	6 W	20 W
	K3	1.8 Ω	Kanthal ^1^	Cotton	K3	10 W	14 W
Eleaf	GS Air	1.5 Ω	Kanthal	Organic cotton	GS	8 W	20 W
	Melo III	0.5 Ω	Kanthal	Organic cotton	MIII	30 W	100 W

^1^ Cubis (1.5 Ω), Nautilus, and K3 atomizers are made with Clapton coils—named in honor of Eric Clapton, the famous guitarist—this particular coil design are composed of two wires, a thicker inner core wire (Kanthal) and a thinner outer wire wrapped around the inner wire, similar to an electric guitar string.

**Table 4:** E-liquid consumptions—average consumption (a) standard deviation (Δa) computed according to the whole number of series and experiments for each commercial atomizer

Devices Acronyms	Reference Power (W)	a (mg·puff^−1^)	Δa (mg·puff^−1^)
Cub0.5	21	5.51	0.11
Cub1	15	9.26	0.21
Cub1.5	12	6.70	0.10
Uni	25	11.12	0.56
CLTank	30	10.04	0.94
MC0.5	30	10.47	0.38
MC1.5	15	5.49	0.71
T18	12	10.55	0.39
I-Sub	20	13.67	0.18
Nauti	11	6.51	0.32
K3	11	4.86	0.15
GS	11	6.33	0.24
MIII	35	9.86	1.45

**Table 5:** Power ranges of the studied devices

Notation	Min Range	Max Range	Reference Power
Cub0.5	15 W	30 W	21 W
Cub1	3 W	32 W	15 W
Cub1.5	8 W	24 W	12 W
Uni	20 W	40 W	25 W
CLTank	15 W	50 W	30 W
MC0.5	15 W	35 W	30 W
MC1.5	10 W	26 W	15 W
T18	8 W	18 W	12 W
I-Sub	20 W	40 W	20 W
Nauti	6 W	20 W	11 W
K3	10 W	14 W	11 W
GS	8 W	24 W	11 W
MIII	30 W	50 W	35 W

**Table 6:** E-liquid consumptions—values of the coefficients (b) and (c) and their standard deviations (Δb) and (Δc) in the equation *MEV* = *bP* + c of the different atomizers according to their required ranges of used power (*R*^2^ = coefficient of determination)

Devices Acronyms	b (mg·puff^−1^·W^−1^)	Δb (mg·puff^−1^·W^−1^)	c (mg·puff^−1^)	Δc (mg·puff^−1^·W^−1^)	*R* ^2^	Power Ranges (W)
T18	1.0034	0.1982	−3.3132	2.5911	0.9277	8–18
Cub1	0.9918	0.0274	−6.7580	0.5203	0.9962	9–27
Cub1.5	1.0810	0.0517	−7.0054	0.7569	0.9932	8–20
MC1.5	1.1728	0.1149	−6.6360	1.6522	0.9905	10–26
Nauti	1.1070	0.0523	−4.1599	0.7121	0.9934	6–20
K3	1.1211	0.1060	−5.2475	1.2596	0.9739	10–14
GS	1.1437	0.079	−6.2745	1.161	0.9906	8–24
Cub0.5	0.7402	0.0365	−8.6700	0.8411	0.9904	15–30
Uni	0.761	0.1176	−5.9537	4.1421	0.9767	30–50
CLTank	0.5190	0.0037	−6.9404	0.1178	0.9999	15–50
MC0.5	0.6827	0.0157	−4.4723	0.4080	0.9984	15–35
I-Sub	0.9115	0.0529	−7.3297	1.4845	0.9931	20–40
MIII	0.7463	0.0297	−13.1439	1.2063	0.9953	30–50
